# Assessment of Entomopathogenic Fungi Activity from the Fiocruz Amazônia Collection in *Anopheles aquasalis* Mosquitoes †

**DOI:** 10.3390/jof11060464

**Published:** 2025-06-18

**Authors:** Natalia Stefany Pereira, Camila Fabbri, Kemily Nunes Moya, Ana Carolina Monteiro Ferreira, Francy’s Sayara Andrade, Rosa Amélia Santana, Claudia Maria Ríos-Velásquez, Priscila Ferreira de Aquino, Stefanie Costa Pinto Lopes

**Affiliations:** 1 Instituto Leônidas & Maria Deane, Fiocruz Amazônia, Manaus 69057-070, AM, Brazil; 2 Fundação de Medicina Tropical Dr. Heitor Vieira Dourado, Manaus 69040-000, AM, Brazil

**Keywords:** malaria, *Plasmodium* spp., entomopathogenic fungi, scanning electron microscopy (SEM)

## Abstract

Malaria remains a public health issue across the world. Different methods have been analyzed to achieve this disease’s elimination, such as the vector control of *Anopheles* spp. Control strategies include the use of different classes of insecticides, although the accelerated evolution of vectors resistant to them makes the development of alternative control methods necessary. Therefore, entomopathogenic fungi have been considered to be promising biopesticides, given that they are safe for human beings and the environment. This study aimed to evaluate the entomopathogenic activity of fungi collected in the Amazon Rainforest against adult female *Anopheles aquasalis* mosquitoes. Females were exposed to four different species of fungi and observed daily to evaluate their survival rate. Also, fungi species’ behavior was analyzed through scanning electron microscopy (SEM). Those exposed to *Trichoderma harzianum* and *Penicillium citrinum* had their survival rate reduced. SEM confirmed the development of fungi on the mosquitoes after 48 h. The findings suggest that the entomopathogenic potential of the fungi used in this study should be considered, given the reduction in the survival rate of *Anopheles aquasalis* mosquitoes.

## 1. Introduction

Malaria remains a public health issue, with 263 million cases in 83 countries in 2023 [1]. Considering the goal of malaria elimination worldwide, researchers have been committed to search new strategies for vector control [2]. Insecticides are widely used as a vector control for malaria. However, their effectiveness has been threatened due to the evolution of mosquitoes resistant to them [3]. Therefore, new strategies tackling the mosquitoes’ resistance against insecticides are essential [4]. One of the strategies used is the use of entomopathogenic fungi, organisms that consist of a wide range of morphologically, phylogenetically, and ecologically diverse fungal species that have evolved to control insects, which can cause their death or even interfere in the reproduction and feeding [5]. Their mode of action involves a four-step process: adhesion of conidia to the insect cuticle, penetration mediated by degradative enzymes, colonization of internal tissues, and finally, sporulation, allowing dissemination to new hosts [6,7,8]. These microorganisms belong to various fungal phyla and exhibit significant ecological, morphological, and phylogenetic diversity. Within entomopathogenic fungi, species of *Penicillium* spp. and *Trichoderma* spp. have shown potential in reducing the survival of different *Anopheles* spp. species [9,10]. Studies indicate that these fungi can act at various mosquito life stages, being particularly effective in the larval and adult phases. Additionally, factors such as mosquito species, developmental stage, fungal concentration, and environmental conditions influence infection effectiveness [11,12].

In this context, this study aimed to assess the effect of entomopathogenic fungi on the survival of *A. aquasalis* adult female mosquitoes. The findings provide valuable insights that could be applied in control programs designed to reduce the survival of this key malaria vector, providing knowledge that can be tested in control programs aiming to reduce the vector’s survival. This article is a revised and expanded version of a paper entitled Atividade entomopatogênica de fungos da coleção da Fiocruz Amazônia em mosquitos *Anopheles aquasalis*, which was presented at XVII Reunião Nacional de Pesquisa em Malária, Belém, PA, Brazil, 8 November 2024 [13].

## 2. Materials and Methods

### 2.1. Anopheles Aquasalis Colony

*Anopheles aquasalis* adult females were obtained from a well-established colony in Unidade de Entomologia Nelson Ferreira Fé (UENFF) at Fundação de Medicina Tropical Dr. Heitor Vieira Dourado (FMT-HVD) in Manaus, AM, Brazil, according to the previously established protocol. The colony were maintained at a constant temperature (24 to 26 °C) and relative humidity (70 to 80%). Mosquito larvae were hatched in salty water at room temperature and fed with commercial fish food (TetraMin^®^, Blacksburg, VA, USA). Adult females of *An. aquasalis* aged 3 to 6 days old and deprived of the 10% sucrose solution 24 h before each assay were used in the subsequent experiments [14].

### 2.2. Fungi Species

The selected species were obtained from *Coleção de Fungos da Amazônia* (CFAM) of Instituto Leônidas & Maria Deane—ILMD/FIOCRUZ Amazônia in Manaus, Amazonas, Brazil. The selected fungi were: *Penicillium citrinum* (CFAM 157), *Penicillium oxalicum* (CFAM 1311), *Trichoderma virens* (CFAM 252), isolated from the soil of the Mamirauá Reserve (Amazonas, Brazil) and *Trichoderma harzianum* (CFAM 1308), isolated from decomposing vegetation and soil in the Lago do Limão Rural Community, Iranduba also in Amazonas, Brazil.

### 2.3. Fungal Culture

**Figure 1:**
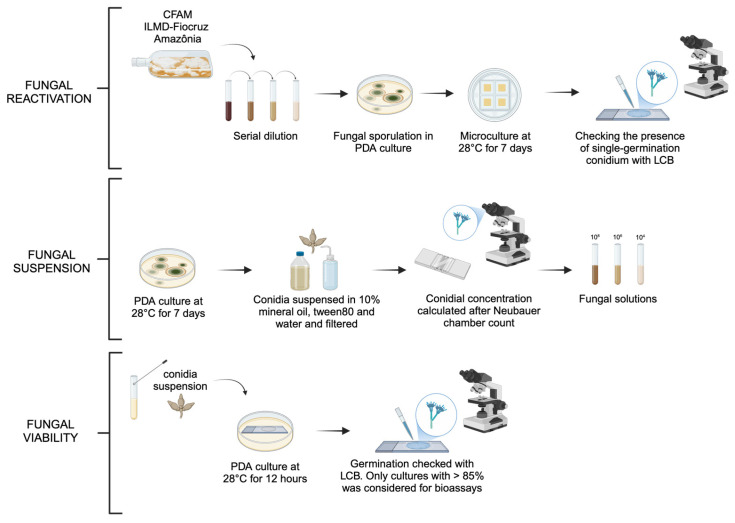
Representative scheme of reactivation, suspension and viability of fungal species investigated. This figure was cin created in BioRender. https://BioRender.com/8yieg0l (accessed on 3 April 2025).

The microorganisms were reactivated in Potato Dextrose Agar (PDA) (KASVI, Weissópolis, PR, Brazil) medium for seven days at 28 °C. For this study, each fungal species was subjected to serial dilution (10^−1^, 10^−2^, 10^−3^, 10^−4^, 10^−5^) to obtain colonies derived from a single conidium [15]. Microculture was performed to confirm each species by optical microscopy (400×), with slide staining using Lactophenol cotton blue (LCB) (Sigma-Aldrich^®^, Darmstadt, Hesse, Germany) (Figure 1) [16].

### 2.4. Preparation and Viability of Fungal Suspensions

Each fungal isolate was cultured in 20 mL of PDA medium for 7 days at 28 °C in a BOD incubator. After growth, conidia were suspended in sterile distilled water with 0.05% Tween 80 (LabsSynth^®^, São Paulo, SP, Brazil), filtered through sterile gauze, and quantified using a Neubauer chamber, which were analyzed in an optical microscope (400×). The concentration was determined using CALIBRA^®^ software (version 1.2). Conidial suspensions (5 mL) were prepared at concentrations of 1 × 10^4^, 1 × 10^6^, and 1 × 10^8^ conidia/mL, containing sterile distilled water with 0.05% Tween 80, 10% mineral oil (RIOQUÍMICA^®^, São José do Rio Preto, SP, Brazil), and 1% Tween 80 (LabsSynth^®^) [10]. The control formulation followed the same composition, except for the absence of conidia (Figure 1).

To ensure the quality of the suspensions used in the bioassays, conidial germination rates were previously assessed on sterile slides coated with PDA medium. On each slide, 20 μL of conidial suspension was deposited in three marked areas and incubated at 28 °C for 12 h inside Petri dishes containing moistened cotton to maintain humidity. After incubation, conidia were stained with LCB, covered with a coverslip, and analyzed under an optical microscope (400×). The viability of the isolates was determined by counting at least 200 germinated conidia per area [17]. Only those with >85% germination was used in bioassays.

Five milliliters of fungal solutions with the desired conidia concentration or control conditions (10% of mineral oil, 1% of tween 80 and distilled water only) were applied by pipetting 20 μL drops throughout the entire filter paper area (80 g/m^2^), which was sterilized before use. The filter paper was left to dry for 30 min at 25 °C in a laminar airflow chamber (Figure 1).

### 2.5. Evaluation of Entomopathogenic Activity of Each Selected Fungi: Survival

The evaluation of mosquito survival was based on the methods of Mnyone et al. [12], with some modifications. To evaluate the entomopathogenic activity of each selected fungus, the impregnated filter paper was fixed in each plastic cage (35 cm diameter × 10.5 cm height) covered with a fine net tissue, preventing mosquitoes from escaping outside. Then, 100 *A. aquasalis* females were transferred to four cages: control cages (filter paper impregnated only with diluent) and cages with filter paper impregnated with 1 × 10^4^, 1 × 10^6^ or 1 × 10^8^ conidia/mL. The mosquitoes were exposed for 24 h. To evaluate survival, they were removed from the cages and transferred to clean ones. Deaths were recorded daily by removing dead mosquitoes from the cage to prevent microorganism growth, until day 25 post-exposure. All mosquitoes used in this experiment were maintained in controlled environmental conditions at 26 °C temperature and humidity between 70% and 80%. Also, mosquitoes were sugar-fed daily with cottons containing a 10% glucose solution [18].

### 2.6. Confirmation of Fungal Species

Since the mosquitoes are not sterile, nor is the environment in which they are maintained, after the evaluation of entomopathogenic activity, mosquitoes from both groups (exposure and non-exposure/control) were checked for the fungus species 56 h post-exposure. To obtain this information, five females were separated and added to a microtube containing 5% Tween80 with vigorous shaking (vortex) to resuspend the fungal spore or mycelium fragments. This suspension was diluted (1:1), and 10 µL was inoculated onto PDA medium supplemented with 0.05% chloramphenicol. After two days, a single germinated conidium was transferred to the same medium. Then, after seven days, a microculture technique was performed to confirm the fungal species. A slide was produced, and the fungal morphology was visualized under optical microscopy (200× and 400× magnification) [19].

### 2.7. Scanning Electron Microscopy

**Figure 2:**
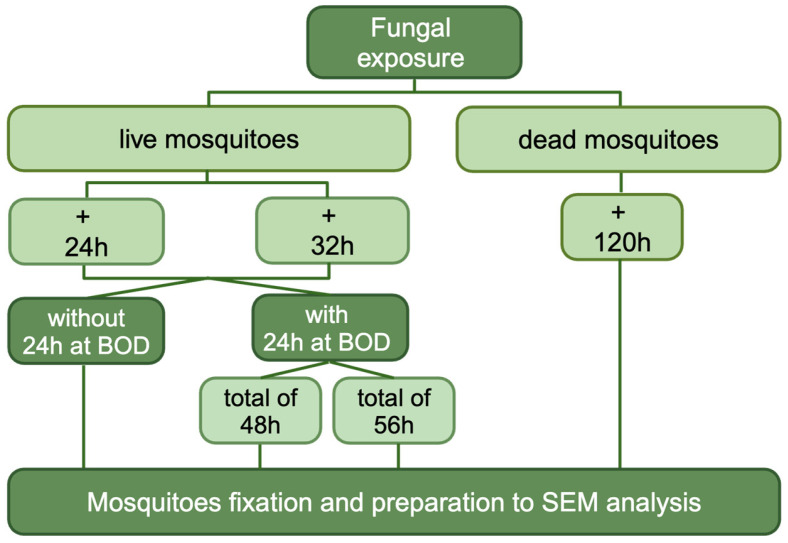
A representative scheme of the mosquito collection for the SEM analysis. This figure was created in BioRender. https://BioRender.com/djpabfb (accessed on 3 April 2025).

The scanning electron microscopy (SEM) was performed to visualize and evaluate the interaction between fungi and insects. For this analysis, several conditions were tested: (1) without culturing the insect in a BOD incubator and collecting the insects at two different times: (1.1) 24 h after the fungus exposure and (1.2) 32 h after the fungus exposure; (2) with culturing the insect in BOD incubator at 28 °C for 24 h, in a Petri dish containing 2% agar growth medium, dissolved only in water, without added nutrients, and collecting the insects after 24 h and 32 h post the fungus exposure. Additionally, the dead mosquitoes were collected 5 days (120 h) after exposition (Figure 2). For each fungus, only the highest concentration and its control groups were selected for SEM [20]. Between 2 and 5 females were collected and analyzed for each condition. The samples were collected in 2 mL microtubes with 4% of buffered paraformaldehyde (4% PFA) recently prepared in Phosphate-buffered saline (PBS), distilled water, and sodium hydroxide, to reach a pH of ~7.2. After that, it was washed five times with a Sodium Cacodylate buffer solution 0.2 M, with a pH of 7.2, and post-fixed in Osmium tetroxide 1% and Potassium Ferrocyanide 0.8% (1:1 proportion). Then, the samples were once again washed in Sodium Cacodylate in the same conditions, and dehydrated in graduated ethyl alcohol series (30%, 50%, 70%, 80%, 90% and 100%) for 20 min in each concentration. After that, samples were dried in Automated Critical Point dryer Leica EM CPD300 (Leica^®^, Wetzlar, Gießen, Germany) and metallized in gold for 2 min with JEOL’s Smart Coater. Electromicrography was performed in JEOL JSM-IT500HR (JEOL Ltd., Tokyo, Japan) [21].

### 2.8. Data Analysis

Survival analyses were calculated using the Log-rank (Mantel–Cox) test comparing all tested groups with control group. *p*-values ≤ 0.05 were considered significant. All statistical analyses were performed in GraphPad Prism^®^ software (Prism 9.5.1; GraphPad Software Inc., Boston, MA, USA).

## 3. Results

### 3.1. Effect of Entomopathogenic Fungi on Anopheles aquasalis Mosquito Survival

*P. citrinum*, *P. oxalicum* and *T. harzianum* were evaluated intwo independent bioassays, while *T. virens* was evaluated in three independent bioassays. *P. citrinum* and *T. harzianum* disclosed entomopathogenic activity, which resulted in *A. aquasalis* survival reduction 24 h after exposure in at least one of the evaluated concentrations. *P. oxalicum* and *T. virens* had non-reproducible results regarding the mosquitoes’ survival. 

**Figure 3:**
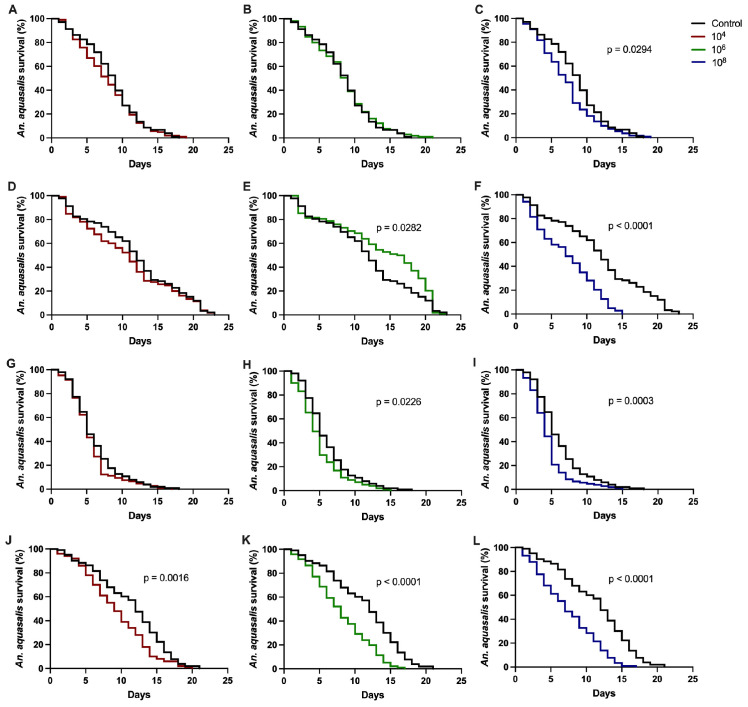
*Anopheles aquasalis* survival after exposure to *P. citrinum* at 1 × 10^4^ (**A**,**D**), 1 × 10^6^ (**B**,**E**), and 1 × 10^8^ (**C**,**F**) concentrations and *T. harzianum* at 1 × 10^4^ (**G**,**J**), 1 × 10^6^ (**H**,**K**), and 1 × 10^8^ (**I**,**L**) concentrations. Each fungus species was tested in two independent bioassays. *p*-values < 0.05 (Mantel–Cox test) are showed in graphics (**C**,**E**,**F**,**H**–**L**).

Exposure to *P. citrinum* significantly reduced mosquito survival in both assays at the highest concentration, compared to the control group. (Table 1 and Figure 3C,F). Moreover, in bioassay 2, we observed a difference between the control group and the group exposed to concentration 1 × 10^6^ (*p* = 0.0282) (Table 1), but the survival of the control was lower, which invalidates the *P. citrinum* entomopathogenic activity in bioassay 2 (Figure 3D).

In *T. harzianum*, the first bioassay exhibited entomopathogenic activity in 1 × 10^6^ and 1 × 10^8^ concentrations (Table 1 and Figure 3H,I), while the second bioassay exhibited it in all the tested concentrations (Table 1 and Figure 3J–L).

### 3.2. Confirmation of Fungal Species

To ratify the species investigated in this study, a confirmation of the fungus’ species found in the mosquito after exposure was performed through protocol according to Jaber et al. [19].

**Figure 4:**
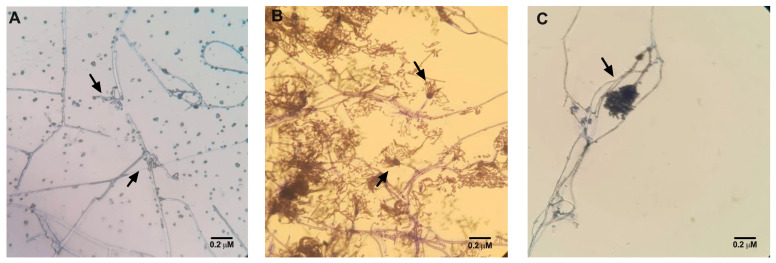
Fungi species confirmation assay after mosquito exposure in slides stained with LCB and observed in optical microscopy ((**A**,**B**) 200× and (**C**) 400× magnification). (**A**) *T. harzianum* from a microculture derived from a mosquito exposed to the same fungus species at 1 × 10^8^ concentration. (**B**) *P. citrinum* control group contaminated with different species of fungus such as *Aspergillus* spp. (**C**) *Aspergillus* spp. contamination in a microculture derived from a mosquito exposed to *P. citrinum* at 1 × 10^8^ concentration. The black arrows point to fungi’s fruiting bodies (conidia).

In the *T. harzianum* bioassays, no other species of microorganism was identified in the mosquitoes exposed to the fungus, except the fungus itself (Figure 4A). Also, in all bioassays, no presence of fungi was found in the control group. On the other hand, the presence of other fungi species, such as *Aspergillus* spp., besides those investigated in this study, was observed in the mosquitoes not exposed—control group (Figure 4B) and exposed to the *P. citrinum* (Figure 4C).

### 3.3. Scanning Electron Microscopy (SEM)

The presence of all fungi species was revealed mainly in the anopheline’s legs (femur and tibia). Table 2 reveals the presence of the fungi species at the time of *Anophele*’s collection and the conditions (incubated in BOD incubator or not) before the fixation with 4% PFA.

**Figure 5:**
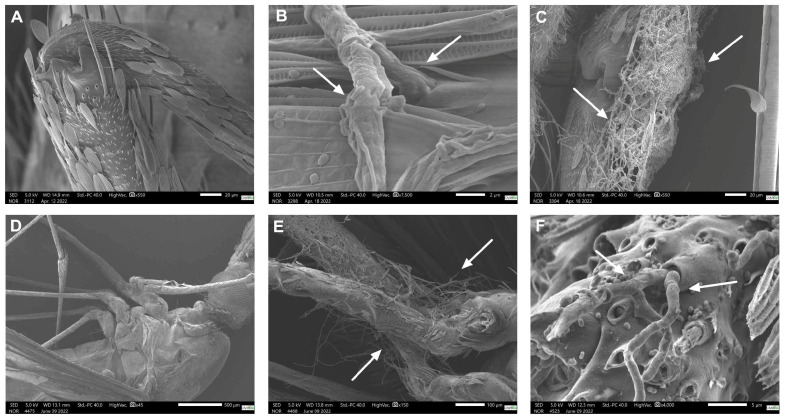
Scanning electron microscopy (SEM) of mosquitoes exposed to *P. citrinum* and *T. harzianum*, both at 1 × 10^8^ concentration. (**A**,**D**) Control group without fungus presence. (**B**,**C**) Parts of mosquitoes exposed to *P. citrinum* collected after 24 h and 32 h, plus 24 h in BOD, respectively. (**E**,**F**) Parts of mosquitoes exposed to *T. harzianum* collected after 24 h and 32 h plus 24 h in BOD, respectively. The white arrows point to fungi’s hyphae and conidia.

In *P. citrinum*, the presence of fungi was observed in different scenarios as described in Table 2. Only the mosquitos exposed for 32 h without the BOD incubation showed hyphae invading the interior of the pore in the leg region (tibia). However, when the mosquitoes were incubated at 28 °C in a Petri dish containing 2% agar growth medium, dissolved only in water, without nutrients, the presence of fungi was observed at both times (24 h and 32 h), as demonstrated in Figure 5B,C, respectively. Figure 5A shows a mosquito from the control group without any presence of fungus.

Regarding *T. harzianum* exposure, no fungi were observed in mosquitoes without a BOD incubation at all collection times. But like in *P. citrinum*, hyphae were seen in both exposure times in the mosquitoes with BOD incubation (Figure 5E,F). Figure 5D shows a control group mosquito without any fungus’ presence.

## 4. Discussion

In this study, the survival of *Anopheles aquasalis* exposed to different fungi species was studied. Similar results were found in other studies, where the survival of African anopheline mosquitoes was significantly reduced by exposure to high concentrations of *M. anisopliae* and *B. bassiana* [12,22,23]. Although there are differences between these studies in relation to the fungal specimen, the oil formulation, the target species and the bioassay protocols, a direct and positive relationship was observed between the concentration of conidia and mortality in all of them. In addition to this information, Mnyone et al. [12]. demonstrated that lower conidia concentrations and short exposure times can result in small infection doses that can be fought by immune responses from mosquitoes, such as melanization, encapsulation, and phagocytosis of the blastopores of invasive fungi, which can be overcome when insects are exposed to high concentrations [12].

The entomopathogenic activity of *T. harzianum* occurs probably due to a combination of enzymes. This fungus secretes extracellular enzymes, such as chitinase and laminase, which restricted, for example, the growth of *Pythium ultimum*, a cosmopolitan fungus which is pathogenic to several plants, by up to 77% [24,25]. Also, in a recent study, the crude chemical constituents extracted from *T. harzianum* were evaluated not only against adult specimen of *A. stephensi*, but also their larvae and pupae. This species demonstrated high mortality rates in all mosquito stages cited [10]. Other species of *Trichoderma asperellum* also demonstrated activity in larvae stages [26]. Also, a recent study demonstrates the larvicidal mechanism of this fungus species [27].

*Penicillium* spp. are fungi largely studied as an entomopathogenic activity against insect larvae, such as the study that tested the entomophatogenic action of *P. citrinum* in *Culex quinquefasciatus* larvae. The result was that the larvae’s survival drastically reduced due to the action mode of mycotoxins, which can cause acute neurotoxicity in this stage [28,29,30]. One study [9] also demonstrated that the magnesium oxide nanoparticles produced from *P. chrysogenum* metabolites can act asa repellent against adult specimens, providing 100% protection for an exposure time between 15 and 120 min. Also, these nanoparticles reduced the survival of the larvae and pupa stages as well [9].

Regarding the SEM, similar results were found in a study by Vieira et al. [20]. The tibia, femur, and anterior thorax regions are known to have many setae, mainly on the abdomen, and their presence can influence the adhesion of conidia to the body [31]. Fungi germs were seen in *P. citrinum*, characterizing the beginning of the germination process. Conidia needs to cross the cuticle and break into the haemocoel to complete its infection process. After the invasion, the fungi can cause the host’s death indirectly through the exhaustion of nutrients and the digestion of internal tissues, or directly through the release of toxins. In most cases, death occurs after the combination of these factors. Therefore, waiting for conidiogenesis to happen in the dead insect is a way to prove the fungi’s effect and the presence of infection [32]. The presence of fungal growth in intersegmental regionscan be explained by the presence of a thin layer of chitin in those regions, which can favor infection through germ tubes. These findings indicate that intersegmental regions—where it is possible to find a big quantity of setae—could be the weak point prone to infection. Similar results were found in *M. anisopliae* [33].

During the experiments, some measures were implemented to prevent external contaminants. Thus, in addition to cleaning the room between procedures, only one bioassay was conducted at a time—and only one species per experiment—to avoid cross-contamination. Nonetheless, we systematically checked for the presence of other fungal species in each bioassay. Therefore, fungal species that are generally found in the environment were observed in *P. citrinum*. This is hypothetically due to a multitude of morphological, physiological, and behavioral parameters related to the insect, which determine its susceptibility to a fungus, in addition to abiotic and biotic environmental factors that also vary in their impact on the virulence of the entomopathogenic fungus. The presence of two or more fungi can cause an additive (synergistic), competitive, and parasitism effect; therefore, the entomopathogenic results found in this species must be considered with caution [34]. Also, the differences between the bioassays can be related to the methodological limitations, given that the technique of exposing mosquitoes to conidia does not guarantee an identical infection, the variation in susceptibility to infection due to the vector’s immune response, and the differences between the viability assays of spores and the germination rate. Therefore, as discussed by Mnyone et al. [12], the effective dose of conidia that really adhere to the mosquito cuticle, invade the hemocoel, and conclude the infection process, is still unknown.

In conclusion, our findings suggest that the entomopathogenic potential of the fungi *T. harzianum* analyzed in this study should be considered after decreasing the survival rate of *An. aquasalis* mosquitoes. This species demonstrated not only a reduction in survival across the three bioassays conducted, but also an entomopathogenic potential at the lowest tested concentration. Additionally, the presence of hyphae was observed in the scanning electron microscopy, and it was confirmed that the test group was contaminated exclusively with the fungus of interest. The entomopathogenic effects of this fungi species from the Brazilian Amazon on adult anophelines was established for the first time, and despite the differences in the ideal concentration and action time, the results presented here can instigate further studies in this topic.

## Figures and Tables

**Table 1:** Comparative *An. aquasalis* survival in mosquitoes exposed to *P. citrinum* and *T. harzianum*.

	Bioassay 1 (*p*-Value)	Bioassay 2 (*p*-Value)
*P. citrinum*		
C ^1^ × 10^4^	NS ^2^	NS
C × 10^6^	NS	0.0282
C × 10^8^	0.0294	<0.0001
*T. harzianum*		
C × 10^4^	NS	0.0016
C × 10^6^	0.0226	<0.0001
C × 10^8^	0.0003	<0.0001

^1^ C = control group. ^2^ NS = not significant. The *p*-values were calculated using Log-rank (Mantel–Cox) test.

**Table 2:** Localization of fungi’s presence (1.5 × 10^8^ concentration) visualized in SEM.

Species	Live Mosquitoes				Dead Mosquitoes
	Without Incubation		With Incubation ^1^		Without Incubation
	24 h	32 h	24 h	32 h	120 h (5 days)
*P. citrinum*	no fungi	tibia	femur	femur and tibia	abdomen
*T. harzianum*	no fungi	no fungi	femur	femur	no fungi
Control group	no fungi presence in all tested conditions				

^1^ 24 h of incubation at 28 °C in BOD.

## Data Availability

The original contributions presented in this study are included in the article. Further inquiries can be directed to the corresponding author.
